# Pharmacological Correctors of Mutant CFTR Mistrafficking

**DOI:** 10.3389/fphar.2012.00175

**Published:** 2012-10-05

**Authors:** Nicoletta Pedemonte, Luis J. V. Galietta

**Affiliations:** ^1^Laboratorio di Genetica Molecolare, Istituto Giannina GasliniGenova, Italy

**Keywords:** cystic fibrosis, CFTR, trafficking defect, drug discovery, chloride channel

## Abstract

The lack of phenylalanine 508 (ΔF508 mutation) in the cystic fibrosis (CF) transmembrane conductance regulator (CFTR) Cl^−^ channel represents the most frequent cause of CF, a genetic disease affecting multiple organs such as lung, pancreas, and liver. ΔF508 causes instability and misfolding of CFTR protein leading to early degradation in the endoplasmic reticulum and accelerated removal from the plasma membrane. Pharmacological correctors of mutant CFTR protein have been identified by high-throughput screening of large chemical libraries, by *in silico* docking of virtual compounds on CFTR structure models, or by using compounds that affect the whole proteome (e.g., histone deacetylase inhibitors) or a single CFTR-interacting protein. The presence of multiple defects of the CFTR protein caused by the ΔF508 mutation and the redundancy of quality control mechanisms detecting ΔF508-CFTR as a defective protein impose a ceiling to the maximal effect that a single compound (corrector) may obtain. Therefore, treatment of patients with the most frequent CF mutation may require the optimized combination of two drugs having additive or synergic effects.

## Introduction

Cystic fibrosis (CF), one of the most common inherited diseases (∼1/3000 in Caucasian populations), is caused by mutations in the CF transmembrane conductance regulator (*CFTR*) gene, which encodes for a cAMP-regulated chloride channel expressed at the apical surface of epithelial cells in the airways, intestine, pancreas, and other organs. Defective Cl^−^ secretion, arising from CFTR mutations, causes a multi-organ disease. In the airways, impaired mucociliary clearance favors recurrent bacterial infection and severe lung damage.

The CFTR protein is composed of five distinct domains: two membrane-spanning domains (MSD1 and MSD2), each having six segments that completely cross the phospholipid bilayer and contribute to the formation of the hydrophilic channel through which anions are transported; two nucleotide-binding domains (NBD1 and NBD2) that are exposed to the cytosol and participate in ATP binding and hydrolysis; a regulatory domain (R) whose phosphorylation regulates channel gating (Riordan, 2005).

The most frequent mutation among CF patients is the ΔF508 mutation, affecting a phenylalanine residue residing in NBD1. Its frequency varies geographically, ranging from about 50% in southern Europe to 70–90% in northern Europe and North America (Bobadilla et al., 2002). Because of its high frequency and severity it has a high priority as a therapeutic target.

Just a few years after the discovery in 1989 of the CF causative gene, the ΔF508 mutation was found to affect the expression and function of the CFTR protein in different ways (Riordan, 2008). The most severe defect consists of a strongly decreased ability to mature and to traffic from the endoplasmic reticulum (ER) to the plasma membrane (PM). The mutant protein is detected by cell quality control (QC) mechanisms as being defective and is degraded by the ubiquitin/proteasome system (Younger et al., 2006; Riordan, 2008). However, it was also found that degradation of the ΔF508-CFTR protein can be reversed by incubating cells at low temperature or with high concentrations of chemical chaperons such as glycerol (Denning et al., 1992; Sato et al., 1996). These experiments demonstrated that the trafficking defect associated with the ΔF508 mutation is correctable, proof of concept for the development of pharmacotherapy using small molecules that correct the basic defect.

However, rescue by low temperature or overexpression also revealed that ΔF508 causes additional defects. First, electrophysiological experiments, particularly patch-clamp recordings, showed that channel activity is significantly reduced by the mutation (Dalemans et al., 1991; Haws et al., 1996). Despite a strong elevation in cytosolic cAMP (CFTR is physiologically activated by cAMP-dependent phosphorylation), open channel probability was approximately one-third of the wild-type protein (Haws et al., 1996). Second, the ΔF508-CFTR protein has a reduced half-time in the PM due to accelerated internalization and degradation (Lukacs et al., 1993; Riordan, 2008).

This type of information evidenced the difficulty in rescuing ΔF508-CFTR expression and function because of the possible requirement of multiple drugs to address the different defects. In particular, it was found that maneuvers that were able to improve trafficking did not affect the channel gating defect and vice versa. Therefore, pharmacotherapy of ΔF508 probably has to be based on the combination of two different types of drugs, generically named *corrector* and *potentiator*, in order to address the trafficking and gating defects respectively (Verkman and Galietta, 2009).

The search for CFTR potentiators has been particularly successful. Campaigns of high-throughput screening and other approaches have identified a plethora of active compounds (Verkman et al., 2006; Verkman and Galietta, 2009). Notably, CFTR potentiators not only increase the activity of ΔF508-CFTR but also of other CFTR mutants with even more severe gating defects. One of these potentiators, VX-770, identified by Vertex Pharmaceuticals (Van Goor et al., 2009) has been particularly successful in clinical trials in patients with G551D (Ramsey et al., 2011), a mutation characterized by very low channel activity but with normal protein trafficking. The drug (named Kalydeco) has been recently approved by the FDA to treat G551D patients.

The search for CFTR correctors has been more difficult and less successful compared to that for potentiators. However, the good results obtained with VX-770 demonstrates that pharmacotherapy of the basic defect in CF is feasible. This represents a formidable driving force for academic laboratories and industry involved in the search of ΔF508 correctors. In the following sections we will summarize the results obtained so far using different approaches and define possible strategies for the future.

## High-Throughput Screening for ΔF508 Correctors

**Figure 1:**
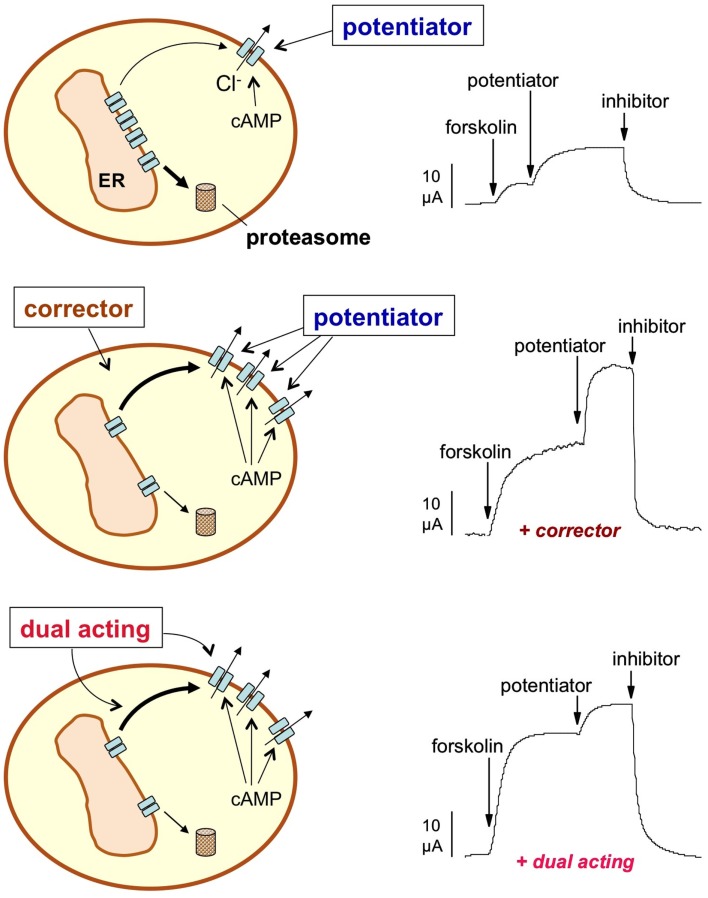
**Pharmacological rescue of ΔF508-CFTR**. The activity of ΔF508-CFTR in the plasma membrane (PM) may be increased by long-term treatment with a corrector, a small molecule that rescues ΔF508-CFTR from the endoplasmic reticulum (ER) and/or increases the half-time of the protein in the PM. The effect of a corrector can be evaluated at the functional level by various technologies such as by directly measuring Cl^−^ currents with electrophysiological techniques (e.g., by short-circuit current recordings shown in the Figure). CFTR activity is first triggered with a cAMP-elevating agent (forskolin) and then further increased with a potentiator, a compound that corrects the intrinsic channel gating defect caused by ΔF508. Finally, a CFTR inhibitor is used to measure the total Cl^−^ current dependent on CFTR. Incubation with a corrector enhances the total current (middle trace) due to the increase in the number of CFTR channels in the PM. A dual-acting compound (bottom trace) not only increases the total current but also the fraction of the current that is elicited cAMP alone thus minimizing the requirement for a potentiator.

In the absence of indications about specific drug targets to rescue ΔF508-CFTR, the most promising and straightforward approach was the screening of large small molecule libraries using functional or biochemical assays. The rationale for this type of approach was that the rescue of the mutant protein from the ER, resulting in increased targeting to the PM (Figure 1), could be measured as an increase in CFTR-dependent anion transport or by directly detecting the CFTR protein on the cell surface with an antibody.

### Verkman project

The first report on the identification of ΔF508- CFTR correctors by screening a very large collection (150,000) of small molecules was published in 2005 by Verkman and collaborators (Pedemonte et al., 2005). The screening assay utilized Fischer rat thyroid (FRT) epithelial cells co-expressing ΔF508-CFTR and the yellow fluorescent protein (YFP) halide indicator YFP-H148Q/I152L in a 96-well microplate format. FRT cells were first used by Sheppard et al. (1994) to study CFTR function. Subsequently, we found that FRT cells are highly useful to identify CFTR pharmacological modulators (Galietta et al., 2001; Zegarra-Moran et al., 2002). First, untransfected FRT cells have negligible levels of anion transport. Therefore, the activity of mutant CFTR after stable expression is not contaminated by endogenous Cl^−^ channels. Second, FRT cells strongly attach to the cell culture support thus resisting all procedures required by high-throughput screening (e.g., cell washings and compound addition). Finally, FRT cells are suitable for a series of electrophysiological assays such as short-circuit current and patch-clamp recordings.

To identify correctors, FRT cells were incubated for 24 h with compounds, washed, and then stimulated acutely with a cocktail of a cAMP agonist plus genistein as a potentiator. CFTR activity in the cell membrane was calculated from the rate of YFP fluorescence quenching caused by extracellular addition and therefore influx of iodide. The study led to the identification of five classes of ΔF508-CFTR correctors (Pedemonte et al., 2005). In particular, two classes of molecules appeared as the most interesting. Class 4 correctors act by improving folding efficiency and by stabilizing immature (core-glycosylated) ΔF508 protein. It is reasonable to assume that the target of class 4 correctors resides in the ER QC system. Instead, class 2 correctors increase the residency time of the mutant protein in the PM, suggesting that the mechanism of action involves the peripheral QC system that targets ΔF508-CFTR toward lysosome-mediated degradation. However, only class 4 correctors, in particular corr-4a, showed efficacy on primary bronchial epithelial cells (Pedemonte et al., 2005). The extent of rescue in these cells was relatively small, with maximal CFTR activity being only 8% of that measured in non-CF cells. Further studies on class 2 compounds identified a particular set, aminoarylthiazoles (AATs), with an interesting dual activity. These compounds improve ΔF508 trafficking as well as channel gating thus reducing the requirement of a potentiator (Figure 1). Interestingly, the effect of AATs on gating was not that of a classical potentiator since it required several hours of treatment. Despite being effective in several cell lines expressing ΔF508, AATs did not reach a significant activity in primary bronchial epithelial cells (Pedemonte et al., 2011).

### Vertex compounds

In addition to the potentiator VX-770, Vertex Pharmaceuticals has also obtained significant results in the discovery of correctors. The company screened a library of 164,000 chemically diverse drug-like compounds using a cell-based assay of membrane potential on NIH-3T3 cells expressing ΔF508-CFTR (Van Goor et al., 2006). The assay reports ΔF508-CFTR activity as a cAMP-stimulated depolarization in the presence of a Cl^−^ gradient. Screening identified 13 structurally distinct scaffolds with corrector activity, six of which were also active on FRT cells with ΔF508-CFTR. The mechanistic data obtained on the quinazolinone class (i.e., VRT-325) suggest that the compounds act primarily or initially at the level of the ER to facilitate the folding and export of ΔF508-CFTR (Van Goor et al., 2006). More important, the subsequent round of optimization of one of the hits from the primary screening led to the investigational drug VX-809 (Van Goor et al., 2011). This compound appeared to be particularly effective in primary cultures of bronchial epithelial cells from ΔF508 CF patients. In combination with the potentiator VX-770, the corrector elicited a 25% rescue. The efficacy shown *in vitro*, plus the safety and tolerability *in vivo*, have allowed the advancement of VX-809 into clinical trials. However, the efficacy of the drug *in vivo* in ΔF508 patients (Clancy et al., 2012) is significantly lower than that of the potentiator VX-770 in G551D patients (Ramsey et al., 2011). For example, the lowering of chloride concentration in sweat, a good indicator of CFTR activity *in vivo*, was 48 mM for VX-770 in G551D patients and 8 mM for VX-809 in ΔF508 patients. In contrast to the potentiator, the corrector did not improve respiratory function or CFTR activity measured by nasal potential recordings (Clancy et al., 2012). These results highlight the particular difficulty in correcting the trafficking defect of the ΔF508 mutation with respect to the gating defect of G551D.

### Sildenafil analogs and RDR1

Researchers at The McGill University identified novel CFTR correctors from a library of 42,000 compounds, by means of a biochemical high-throughput assay in a 96-well microplate format (Robert et al., 2008). Screening was performed using BHK cells, which stably express ΔF508-CFTR bearing three tandem hemagglutinin (HA) epitope tags in the fourth extracellular loop after amino acid 901. The appearance of ΔF508-CFTR or wild-type CFTR at the cell surface was monitored in a plate reader with an anti-HA antibody and a fluorescent secondary antibody. The study led to the identification of different compounds, in particular the approved drug sildenafil, along with several structural analogs with improved potency, having activity as ΔF508-CFTR correctors. Later on, the same group developed a new assay, based on differential scanning fluorimetry, to identify pharmacological chaperones of ΔF508-CFTR, i.e., compounds that bind and act directly on the mutated NBD1 domain of ΔF508-CFTR. The hits derived from the previous cell-based screen for CFTR correctors were tested by the authors, which identified one compound, the phenylhydrazone RDR1, able to bind to and thermally stabilize purified murine ΔF508-NBD1 *in vitro* (Sampson et al., 2011).

### MPB compounds

A small-scale screening for CFTR activators performed by Becq et al. (1999) using iodide efflux experiments resulted in the description of a class of tricyclic compounds called benzo[c]quinoliziniums or MPB compounds. The compounds MPB-07 and MPB-27 appeared as selective activators of wild-type CFTR in different cell systems. Subsequently, synthesis of new derivatives identified MPB-91 as a potent activator of G551D-CFTR (Derand et al., 2001). Soon after, by studying the ΔF508-CFTR activity and the trafficking by immunofluorescence in freshly isolated native airway epithelial cells from CF patients, the authors realized that treatment of cells with MPB-07 caused dramatic relocation of ΔF508-CFTR to the apical region such that the majority of CF cells showed a pattern similar to that of non-CF cells (Dormer et al., 2001). Further studies demonstrated that benzo[c]quinoliziniums selectively inhibit degradation of the ΔF508 protein, by protecting a proteolytic cleavage site by direct binding to the first cytoplasmic domain of ΔF508-CFTR, thus resulting in increased ΔF508-CFTR trafficking (Stratford et al., 2003).

## Structure-Based Corrector Design

Although high-resolution structural information on full-length CFTR protein is still missing, studies on the structure of CFTR NBD1 and homologous ABC transporters has provided insights into the three dimensional architecture of CFTR (Lewis et al., 2004, 2005; Rosenberg et al., 2011; Lukacs and Verkman, 2012). In native CFTR, NBD1 interfaces with the cytoplasmic loops 4 (CL4) and 1 (CL1) in MSD2 and MSD1, while NBD2 associates with CL2 and CL3 of MSD1 and MSD2 respectively (Lukacs and Verkman, 2012). These interfaces not only transmit the ATP-dependent conformational changes occurring in NBDs to MSDs during channel gating, but also play a crucial role in CFTR biogenesis (Lukacs and Verkman, 2012). Indeed, ΔF508 mutation destabilizes the conformation of MSD1, MSD2, and NBD2, by impairing the assembly of the interface between NBD1 and MSD2/MSD1, resulting in protein misfolding (Lukacs and Verkman, 2012).

### Epix project

Starting from the structural information available for CFTR and other ABC proteins, researchers at Epix Pharmaceuticals performed an *in silico* structure-based screening for ΔF508 correctors utilizing homology models of CFTR (Kalid et al., 2010). After modeling the intracellular region of CFTR, they identified three cavities at inter-domain interfaces: (1) the interface between the two NBDs; (2) the interface between NBD1 and CL4, in the region of the F508 deletion; (3) the multi-domain interface between NBD1 and 2 and CL1, 2, and 4. The working hypothesis was that compounds binding at these interfaces may improve the stability of the protein, potentially affecting the folding yield or surface stability. *In silico* structure-based screening of a focused library of ∼100,000 compounds (extracted from the EPIX in-house database containing ∼4-million unique compounds) highlighted 496 candidate compounds that were tested in functional assays. The study resulted in the identification of 15 novel compounds of diverse chemotypes, active as ΔF508 folding correctors. Interestingly, all the binding sites subjected to screening yielded CFTR potentiators as well as correctors. In addition, several of the chemical series were found to harbor the potential for both types of activities, with small chemical modifications independently modulating the activity as corrector or potentiator. Notably, the study also led to the identification of several compounds with a dual corrector-potentiator activity (dual-acting). According to the authors, this could be due to the fact that they used a CFTR model representing the conducting state of the channel. Stabilizing this state by direct binding of small molecules may increase the open probability of the channel (potentiation), improve the stability of the protein (potentially affecting the folding yield or surface stability of the protein, i.e., correction), or both.

## Hypothesis-Driven Search for ΔF508 Correctors

**Figure 2:**
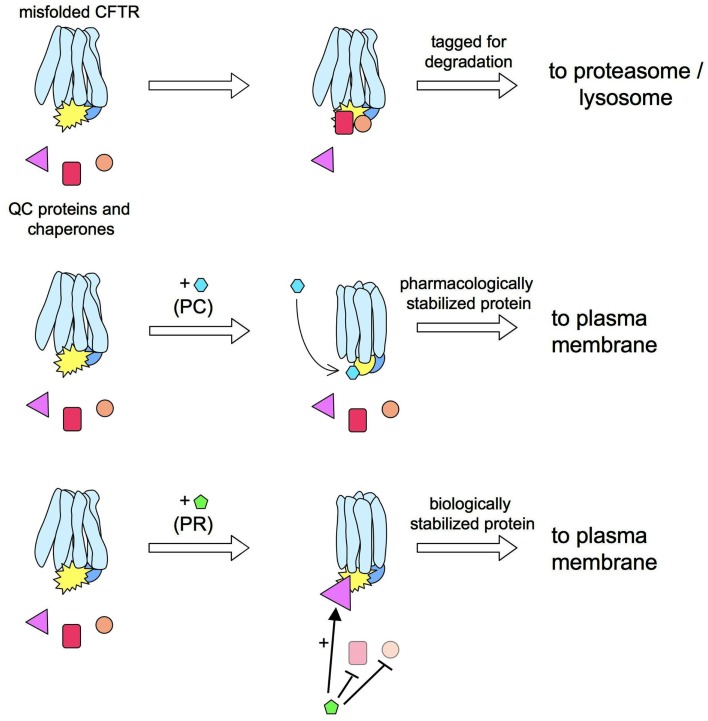
**Pharmacological chaperones vs. proteostasis regulators**. ΔF508-CFTR rescue may be obtained by a pharmacological chaperone (PC) that interacts directly with the mutant protein. For example, a PC may increase the CFTR stability by improving the interaction between CFTR domains. This effect would prevent the detection of ΔF508-CFTR by quality control (QC) proteins thus allowing more protein in the plasma membrane (PM). An alternative approach for ΔF508-CFTR is the use of a proteostasis regulator (PR). These compounds act by globally changing the proteome, or a more restricted group of proteins, to create an environment more benign toward mutant CFTR.

The mechanisms of action of correctors have not been clarified, and it is not known whether they interact directly with CFTR (i.e., acting as pharmacological chaperones) or with other intracellular proteins (Figure 2). However, considering the discrepancies between their effects on heterologous expression systems versus native epithelial cells from CF patients (Pedemonte et al., 2010), it is reasonable to conclude that many correctors do not interact directly with ΔF508-CFTR to favor its folding and stabilization. If that were the case, one would expect an activity that is independent of cell background. Rather, it is probable that many correctors modulate QC mechanisms responsible for mutant CFTR detection and degradation (i.e., they act as “proteostasis regulators”; see Calamini et al., 2011).

Proteostasis regulators (Figure 2) are considered interesting therapeutic agents to treat genetic diseases with protein misfolding defects. Indeed, loss of proteostatic control has been implicated in aging and in multiple disorders of protein misfolding, in which the chronic expression and accumulation of misfolded, oxidized, and aggregated proteins leads to cellular dysfunction. There is increasing evidence that misfolded proteins expressed in diseases of protein conformation are not efficiently counterbalanced by a compensatory induction of cellular stress responses such as the heat shock response and the unfolded protein response (Calamini et al., 2011). Enhancing the activity or increasing the expression of molecular chaperones through genetic techniques or pharmacological manipulation has been shown to restore proteostasis in several disease models (Calamini et al., 2011).

### Modulation of histone acetylation

Histone acetyl transferases (HATs) and deacetylases (HDACs) are enzymes that mediate post-translational acetylation and deacetylation reactions, respectively, of histones, transcription factors, and other cytosolic factors, leading to modulation of transcriptional events during development and in response to environmental changes (Hutt et al., 2010). Researchers at the Scripps Institute (La Jolla, CA, USA), headed by William Balch, evaluated the effect of knocking down single HDACs to address their specific roles in human health and disease (Hutt et al., 2010). The study demonstrated that HDAC7 suppression by siRNA-mediated silencing or with the HDAC inhibitor SAHA, resulted in a substantial increase in stabilization, trafficking, and activity of ΔF508 cell surface chloride channel activity. The authors proposed that the mechanism by which HDAC inhibition may ameliorate CF and possibly other misfolding diseases involves the capacity to create an intracellular environment that is more benign toward misfolded proteins (Hutt et al., 2010). The efficacy of SAHA in primary airway epithelial cells from ΔF508 patients has not been confirmed in other studies (Sondo et al., 2011; Van Goor et al., 2011). This may indicate that the net balance of effects induced by HDAC inhibitors may be significantly affected by experimental conditions.

### Modulation of ER calcium pumps

Experimental evidence suggests that inhibitors of ER calcium pumps correct the ΔF508 trafficking defect through partial inhibition of the interaction between ΔF508-CFTR and calnexin, a ER lectin-like protein that binds monoglucosylated oligosaccharides (Norez et al., 2006a). On this basis, Becq and colleagues hypothesized that by inhibiting the deglucosylation of ΔF508 protein in the ER, glucosidase inhibitors may prevent the interaction of ΔF508-CFTR with calnexin and hence its entry into the degradation pathway (Norez et al., 2006b). To verify this hypothesis, the authors tested two compounds that inhibit ER α-1,2-glucosidase, miglustat (an *N*-alkylated imino sugar also called *N*-butyldeoxynojirimycin), and castanospermine, as well as an inactive imino sugar analog (*N*-butyldeoxygalactonojirimycin). The study demonstrated that miglustat rescues ΔF508-CFTR in human and mice epithelial cells and prevents the interaction of ΔF508-CFTR with calnexin in the ER, suggesting that inhibition of deglucosylation of nascent proteins may be the molecular mechanism of the compound’s effect (Norez et al., 2006b).

## Lessons from Biochemistry and Cell Biology

The improved knowledge over the last few years of the molecular mechanisms involved in CFTR biosynthesis, trafficking, and degradation is helping us understand the consequences of the ΔF508 mutation and the suitability of these mechanisms as therapeutic targets (Lukacs and Verkman, 2012). First of all, it is clear that ΔF508-CFTR is scrutinized by multiple quality control checkpoints both at the level of the ER and the PM. In particular, nascent ΔF508-CFTR is marked early on for degradation in the ER by the ubiquitin ligase RMA1 in combination with Derlin-1 (Younger et al., 2006). At a later stage, when mutant CFTR is fully synthesized, other proteins, such as the ubiquitin ligase CHIP, intervene (Younger et al., 2006). Interestingly, a siRNA-based small scale screening has revealed that CHIP and other proteins involved in ER-associated degradation of CFTR are also important in peripheral QC and affect the half-time of mutant CFTR in the PM (Okiyoneda et al., 2010). Therefore, there is a redundancy of mechanisms responsible for the detection of ΔF508-CFTR as a mutant protein. Another aspect of ΔF508-CFTR is the possibility of trafficking to the PM by an unconventional route. Under particular conditions, such as incubation of cells at low temperature or blockade of ER-to-Golgi transport, ΔF508-CFTR may reach the cell surface in a Golgi-independent way (Gee et al., 2011). The plethora of QC and trafficking mechanisms associated with ΔF508-CFTR explains the different observations reported in various studies. For example, it has been repeatedly reported that the combination of small molecules has additive or synergic effects on ΔF508-CFTR rescue (Pedemonte et al., 2011). This kind of effect may also be obtained by combining a corrector with the silencing of a QC protein. For example, treatment with corr-4a plus silencing of RMA1 led to a 13-fold increase in ΔF508-CFTR maturation (Grove et al., 2009). The additive/synergic effects of drug combinations clearly point to different mechanisms of action. In fact, it was found that corr-4a affects a step downstream of RMA1 (Grove et al., 2009). Another consequence of QC redundancy is the sensitivity to cell background. It is reasonable to hypothesize that the relevance of some mechanisms may change from one cell type to another as we have recently demonstrated (Pedemonte et al., 2011). This has important practical implications: a corrector found by screening in a given cell line may not be effective in another cell type and, particularly, in primary airway epithelial cells.

Different studies indicate that the instability of ΔF508-CFTR arises from two main characteristics: the intrinsic instability of NBD1 and the defective docking of NBD1 to CL4. These results point to two separate defects, both of which need to be corrected. This requirement has been recently demonstrated in two independent studies (Mendoza et al., 2012; Rabeh et al., 2012). High levels of ΔF508 rescue have been obtained only when suppressing mutations have been introduced both in NBD1 and in CL4. The first type of mutation, such as I539T or R555K, increases the stability of NBD1. The second type of mutation, namely R1070W, improves the interaction of NBD1 with CL4 by providing an aromatic group that compensates for the lack of F508.

## Perspectives

In conclusion, the increasing knowledge on ΔF508 is indicating that it may not be possible to fully correct the trafficking defect with a single compound. Several *in vitro* studies point out that a large rescue may be obtained only with a combination of correctors. More effective correctors may be identified by high-throughput screening of compounds with novel and unexplored structure, by exploiting the increasing information available on CFTR structure, or by taking advantage of the identification of important proteins of the CFTR interactome. In this respect, genome-wide siRNA screening could be very useful to identify novel proteins with a high relevance for CFTR QC, trafficking, and regulation. The possible need for two correctors to treat ΔF508 represents a problematic scenario in terms of drug development and clinical testing. However, the concept of drug combination in CF (e.g., a corrector plus a potentiator) is already accepted. The use of two correctors instead of one potentiator and a corrector may be justified if both compounds together elicit a high level of CFTR function. In the near future, novel and effective treatments for the CF basic defect are expected. These advances also represent an important proof of concept and a paradigm for other genetic diseases.

## Conflict of Interest Statement

The authors declare that the research was conducted in the absence of any commercial or financial relationships that could be construed as a potential conflict of interest.
